# Preclinical pharmacokinetics/pharmacodynamics studies defining the role of ethambutol in *Mycobacterium kansasii* lung disease

**DOI:** 10.1128/aac.01447-25

**Published:** 2025-12-19

**Authors:** Tawanda Gumbo, Gunavanthi D. Boorgula, Shashikant Srivastava

**Affiliations:** 1 NASOS Biotech, Dallas, Texas, USA; 2 IMPI Group, Tateguru, Zimbabwe; 3 Division of Infectious Diseases, Department of Medicine, The University of Texas at Tyler School of Medicine 675071, Tyler, Texas, USA; 4 Department of Cellular and Molecular Biology, University of Texas Health Science Center at Tyler 12341, Tyler, Texas, USA; City St George's, University of London, London, United Kingdom

**Keywords:** pharmacokinetics/pharmacodynamics, minimum inhibitory concentrations, hollow fiber system model of *Mycobacterium kansasii *disease, ethambutol-resistance, cavitary concentrations

## Abstract

Rifampin, isoniazid, and ethambutol are the backbone of the regimen used to treat *Mycobacterium kansasii-*complex (MKC) lung disease. However, ethambutol pharmacokinetics/pharmacodynamics (PK/PD) studies to inform on optimal exposure target and clinical dose for MKC are lacking. We performed studies to determine ethambutol minimum inhibitory concentration (MIC), mutation frequency (3× MIC), a PK/PD study using the hollow fiber system model of MKC (HFS-MKC) using the reference ATCC#12478 strain, and Monte Carlo simulation experiments for clinical dose selection and susceptibility breakpoint. We also performed a literature search to generate ethambutol MIC distribution for MKC. First, nine studies were identified with MIC of 587 isolates, and MIC_50_ and MIC_90_ identified as 4 and 16 mg/L, respectively. Second, the ethambutol MIC of the ATCC strain was 8 mg/L, and the mutation frequency was 4.23 × 10^−2^ CFU/mL. Third, in the HFS-MKC, ethambutol failed to kill *M. kansasii* below stasis (*B*_0_), and resistance emerged rapidly. The target exposure was an AUC_0–24_/MIC of 5.47 (95% confidence interval: 1.17–9.77). Fourth, Monte Carlo experiments of 10,000 virtual subjects identified doses of 1,200 and 3,000 mg to achieve or exceed target exposure in 18.21% and 58.57% of patients; and PK/PD MIC susceptibility breakpoints were determined as 2 and 4 mg/L, respectively. Doses >1,200 mg/day may have a higher likelihood of ocular toxicity. The risk of toxicity versus no microbial kill benefit in HFS-MKC suggests the need for better drugs compared to ethambutol in the treatment of MKC lung disease.

## INTRODUCTION

Among the neglected maladies that constitute non-tuberculous mycobacterial (NTM) lung diseases, *Mycobacterium kansasii-*complex (MKC) lung disease is perhaps even more neglected. As a result, treatment guidelines for MKC lung disease were scored as a “conditional recommendation, very low certainty in estimates of effect” (1). The guidelines suggest a combination of rifampin and ethambutol, while the third drug, isoniazid, can be replaced by a macrolide, for at least 12 months (1). Clearly, shorter treatment regimens are required. Moreover, while ethambutol is believed to play the role of suppressing resistance to rifamycins, further evidence is also required as to the contributions of individual drugs to microbial kill if “strong recommendations” are to be made. To achieve this, previously we reported pharmacokinetics (PK)-pharmacodynamics (PD) studies with rifampin and isoniazid contributions, as well as potential roles of rifapentine, tedizolid, omadacycline, clofazimine, and moxifloxacin, in the hollow fiber system model of MKC (HFS-MKC) lung disease (2–8). Here, we report ethambutol PK/PD studies in the HFS-MKC.

Ethambutol is a standard tuberculosis (TB) drug that was repurposed for treatment of MKC lung disease (9). Inclusion of ethambutol in the first 2 months of the anti-TB therapy is recommended in high HIV prevalence settings in case of isoniazid resistance (10). However, using artificial intelligence (AI) approaches in TB patients, we demonstrated that ethambutol has a concentration-dependent sterilizing effect in patients (i.e., kills persistent bacilli) when rifamycin concentrations are low (11). Moreover, in patients with *M avium*-complex lung disease on treatment with a macrolide, treatment with ethambutol was associated with an odds ratio of 18 for sputum conversion versus a macrolide alone, and an odds ratio of 17 after adding both ethambutol and rifampin (12, 13). Furthermore, in *M. vaccae*, ethambutol was shown to increase the permeability of the mycobacterial cell wall to macrolides and rifampin, enhancing their effect (12, 13). This means that the role of ethambutol beyond protecting other drugs to resistance needs to be investigated.

There are important advantages to ethambutol being a repurposed drug; we have a lot of learnings from TB and *M. avium* lung diseases. First, the major clinical presentation of MKC lung disease is cavitary disease; large cavities are associated with higher mortality on treatment (14, 15). Important PK factors such as ethambutol penetration into lung cavities have been extensively mapped in TB patients, and ethambutol’s PK/PD drivers for TB and *M. avium* complex in the HFS (peak concentration [*C*_max_]-to-minimum inhibitory concentration (MIC) in serum of 1.23 or 0.77 in lung cavities) as well as in patients (*C*_max_/MIC > 0.46) are well understood (11, 16–20). In human TB lung cavities, ethambutol concentration-distance gradient into the cavity is characterized as a dynamical system with the sink at cavity center (air-caseum interface) where there is the lowest drug 0–24 h area under the concentration-time curve (AUC_0–24_) but the highest bacterial burden (19, 21–23). Here, we employed PK/PD approaches in the HFS-MKC that took into account concentrations at the center of cavities in patients. We performed Monte Carlo experiments that utilized these concentration gradients for identification of optimal doses. Finally, from a clinical perspective, the HFS-MKC microbial responses have been quantitatively mapped to patients’ sputum responses using category theory and extinction mathematics, so that the microbial kill can be directly translated from this preclinical *in vitro* tool to sustained sputum culture conversion in patients (4, 24–27).

## RESULTS

**Fig 1:**
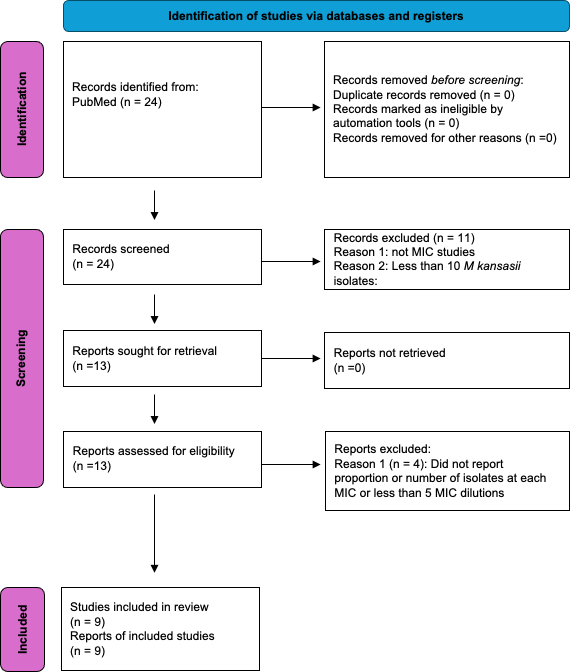
Literature search for ethambutol MIC distribution based on PRISMA guidelines.

The ethambutol MIC of the MKC standard laboratory isolate (American Type Culture Collection ATCC#12478) was 8 mg/L. Based on culturing 4.0 mL of the HFS-MKC inoculum on 20 Middlebrook 7H10 agar plates supplemented with ethambutol 24 mg/L (3× MIC), the mutation frequency was 4.23 ± 1.54 × 10^−2^ CFU/mL or a prevalence of 4.23%. Next, a PubMed search using the medical subject (MeSH) headings “*Mycobacterium kansasii* AND MIC AND ethambutol” was conducted to generate MIC distributions. Figure 1 shows that we identified nine studies that fulfilled the criteria described in detail in Materials and Methods section (28–37). The nine studies are summarized in Table 1. Since all studies employed the broth microdilution method, we combined the MICs of all 587 isolates into a single distribution. Gaussian analysis confirmed that all distributions (including the one for all 587 isolates) shared the mean MIC value of 9.98 mg/L (close to the MIC of 8 mg/L of the ATCC strain), confirming that they could be combined.

**TABLE 1:** Minimum inhibitory concentration (MIC) (mg/L) distributions identified in literature search

Reference	Year	Location	*n*	MIC range	0.25	0.5	1	2	4	8	16	32	64	MIC_50_	MIC_90_
Ahn et al. (34)	1987	Texas, USA	14	1.0–16.0			6	8	0	0	0				
Utrup et al. (33)	1995	Pennsylvania, USA	10	0.25–128.0	0	0	0	0	0	3	7	0	0	16	16
Guna et al. (32)	2005	Valencia, Spain	104	0.5–8.0		2	2	43	54	3				4	4
Litvinov et al. (35)	2018	Moscow, Russian Federation	112	0.5–16		3	1	17	49	17	25			4	16
Bakuła et al. (30)	2018	Poland and the Netherlands	62	1.0–32.0						6	28	28		>16	>16
He et al. (36)	2022	Wenzhou, China	14	0.5–16.0		0	1	7	0	1	5			2	16
Guo et al. (37)	2022	Shanghai, China	60	0.5–16.0		1	2	3	8	16	26	4		8	16
Zhang et al. (28)	2023	Shanghai, China	191	0.5–16.0		2	2	11	57	89	25	5		8	16
Srivastava et al. (29)	2023	Texas and Virginia, USA	20	1.0–64.0			0	3	9	2	1	1	4	4	64
		Total	587		0	8	14	92	177	137	117	38	4		
		Proportion			0	0.01	0.02	0.16	0.30	0.23	0.20	0.07	0.01		
		Cumulative				0.01	0.04	0.19	0.50	0.73	0.93	0.99	1.00	4	16
Relapse and treatment failures															
Ahn et al. (34)	1987	Texas, USA	14	1.0–16.0			2	3	3	4	2				

**Fig 2:**
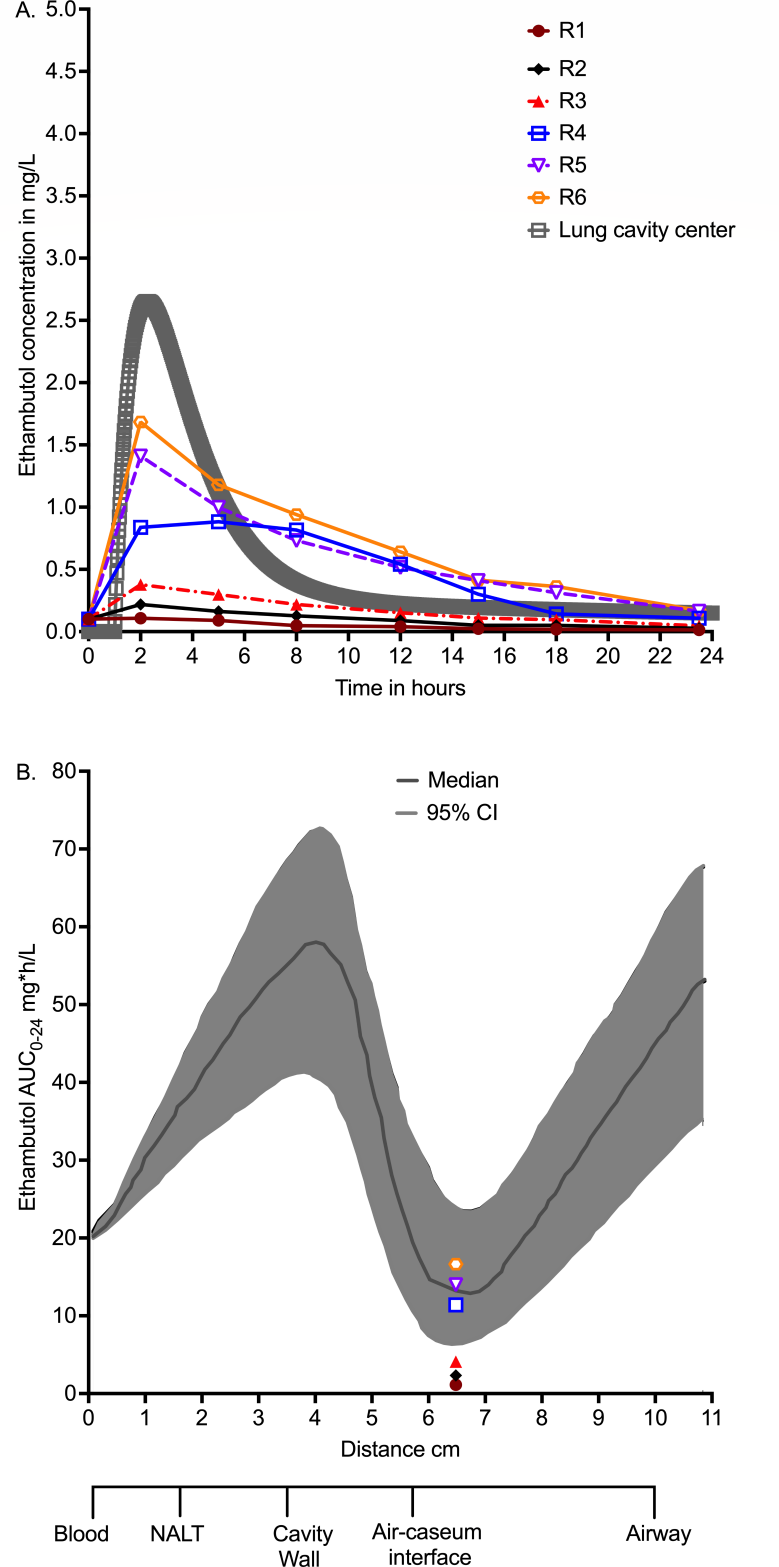
Drug concentrations measured in HFS-MKC and in patients’ lung cavities (19). (**A**) Ethambutol pharmacokinetic model of concentrations measured at the center of lung cavities in patients, overlaid on concentrations measured in the hollow fiber system model of *Mycobacterium kansasii* (HFS-MKC) at different time points. (**B**) Ethambutol 0–24 area under the concentration-time curves (AUC_0–24_) measured at various positions in lung cavities and modeled using dynamical sink models in our previous publication (19), compared to those measured in the HFS-MKC.

Measurement of drug concentrations in HFS-MKC units inoculated with ATCC#12478 revealed the ethambutol concentration-time profiles as shown in Fig. 2A. The non-protein-bound concentrations are compared to those measured in the lungs of patients who received 1,200 mg per day of oral ethambutol in our prior publications (18, 20–22). Based on the concentration-time profiles in the HFS-MKC in Fig. 2A, all six HFS-MKC units (designated R1–R6) had % time above MIC (%T_MIC_) of 0. Figure 2B compares the resultant AUC_0–24_ observed in the HFS-MKC to the AUC-gradient encountered at different positions along the lung cavities of patients on treatment from our previous publications (18, 20–22). These data show that half of the ethambutol AUCs achieved in the HFS-MKC were in the range observed at the air-caseum interface in lung cavities of patients in the past.

The HFS-MKC was an intracellular model in that THP-1 cells were first infected, and then 2.73 × 10^6^ cells/mL were added to the peripheral compartment of each HFS-MKC unit. The THP-1 cells lasted until day 7, after which the counts fell and could not be counted. Thus, the HFS-MKC model tested both intracellular and extracellular bacilli.

**Fig 3:**
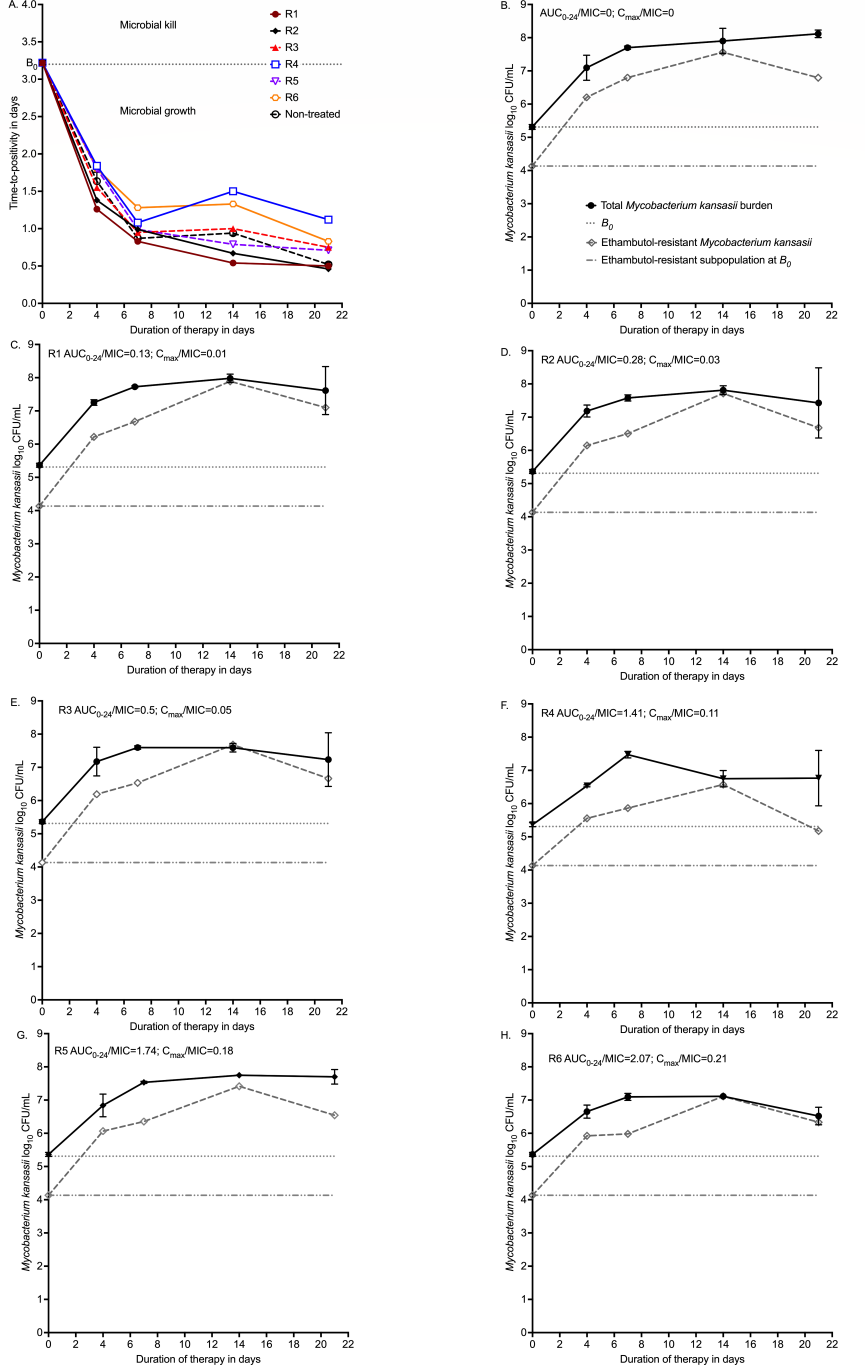
Time-kill and resistance emergence in the HFS-MKC. (**A**) Time-kill curves are shown using time-to-positivity. (**B–H**) Total bacterial burden versus drug-resistant burden by exposure. AUC_0–24_ = 0–24 area under the concentration-time curves; *B*_0_ = bacterial burden at time 0 in the inoculum; *C*_max_ = peak concentration.

Overall, none of the six ethambutol exposures killed intracellular MKC below day 0 bacterial burden (*B*_0_) during the entire 21 days of study, as shown in Fig. 3A. Figure 3A is based on time-to-positivity (TTP) readout in the Mycobacteria Growth Indicator Tube (MGIT) liquid culture system. Figure 3B through G shows the total MKC population versus the ethambutol-resistant subpopulation for each of the treatment regimens (R1–R6), including the non-treated controls. There was a rapid emergence of ethambutol resistance to the monotherapy in all drug-treated HFS-MKC units. Figure 3B shows non-treated controls, and the drug-resistant subpopulation was parallel to the total population, consistent with the 4.23% mutation prevalence. Figure 3C shows that the total population was replaced by the ethambutol-resistant subpopulation by day 14. Figure 3D through F shows basically the same pattern, except that the ethambutol-resistant subpopulation at the end of the experiment on day 21 fell steadily with increasing exposure, with the lowest subpopulation in Fig. 3F at 5.18 log_10_ CFU/mL. Beyond that exposure, the size of the ethambutol-resistant subpopulation began to increase until it replaced the drug-susceptible population by the end of the experiment (day 21) in Fig. 3H.

**Fig 4:**
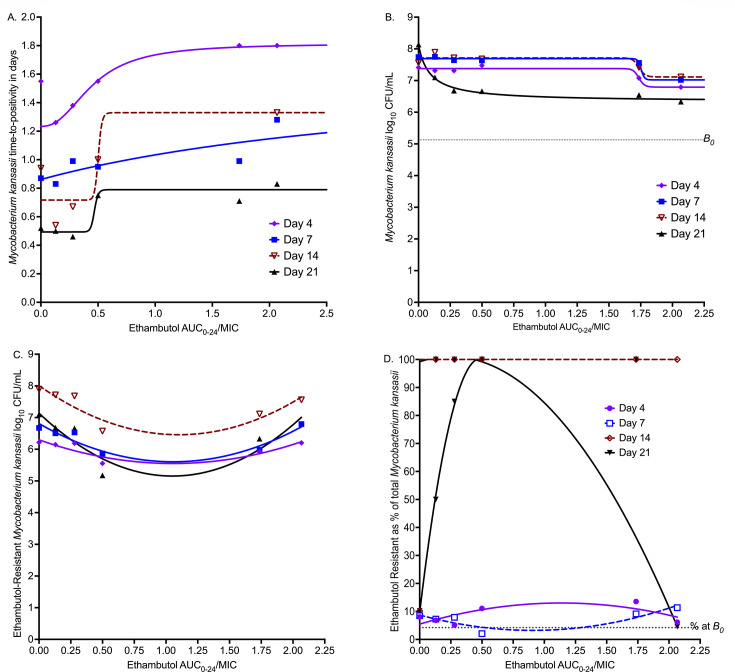
Pharmacokinetic/pharmacodynamic modeling for microbial kill and resistance. (**A**) Total bacterial population based on time-positivity readout versus AUC/MIC exposure. (**B**) Total bacterial population based on CFU/mL readout versus AUC/MIC exposure. (**C**) Ethambutol-resistant population (log_10_ CFU/mL) versus exposure. The symbols are bacterial burden. (**D**) Resistance is shown as % of the total population. % at *B*_0_ is the percentage resistance at *B*_0_, and any proportion above this means resistance amplification. *B*_0_ = bacterial burden at time 0 in the inoculum. Shown are regression curves on different sampling days.

Inhibitory sigmoid *E*_max_ models for each sampling day are shown in Fig. 4A, for MGIT-derived TTP readout, and in Fig. 4B for log_10_ CFU/mL readout. Model parameter estimates for both readouts are shown in Table 2, including EC_80_s (optimal exposure) calculated for each sampling day. Since AUC_0–24_/MIC exposures had better corrected Akaike information criterion scores (38) on each sampling day compared to *C*_max_/MIC and % of time concentration persists above MIC (%T_MIC_), we show results as AUC_0–24_/MIC versus bacterial burden. Table 2 shows that the EC_50_ changed outside 95% confidence intervals (CIs) from sampling day-to-day with a coefficient of variation of 95.60% for the CFU/mL readout (AUC_0–24_/MIC minimum 0.09 and maximum of 3.84), and 81.35% for TTP (AUC_0–24_/MIC minimum 0.45 and maximum 3.84). The EC_80_ was taken as the average of all sampling days and was an AUC_0–24_/MIC of 5.47 (95% CI: 1.17–9.77). Figure 4C shows AUC_0–24_/MIC versus ethambutol-resistant subpopulation as log_10_ CFU/mL, while Fig. 4D shows it as a % of total population modeled using the antibiotic arrow of time model. Figure 4D shows the typical system of “U” curves and inverted “U”s, which changed with sampling time. The exposures associated with suppression of resistance below 4.23% in the *B*_0_ also varied by sampling day.

**TABLE 2:** Inhibitory sigmoid *E*_max_ parameters estimates and standard error for each sampling day

	Day 4	Day 7	Day 14	Day 21
	Estimate ± SE	Estimate ± SE	Estimate ± SE	Estimate ± SE
CFU/mL readout				
*E*_con_ log_10_ CFU/mL	7.45 ± 0.22	7.62 ± 0.04	7.71 ± 0.07	8.14 ± 0.11
*E*_max_ log_10_ CFU/mL	0.62 ± 1.09	0.64 ± 0.09	1.42 ± 1.55	1.81 ± 0.17
*H*	1.60 ± 4.36	40.22 ± 231.9	1.0 ± 1.5	0.98 ± 0.49
EC_50_ (AUC_0–24_/MIC)	3.84 ± 11.42	1.81 ± 0.43	0.97 ± 2.05	0.09 ± 0.03
EC_80_ (AUC_0–24_/MIC)	9.13 ± 27.16	1.87 ± 0.45	3.88 ± 8.2	0.37 ± 0.12
*r*^2^	0.96	0.98	0.90	0.99
Time-to-positivity readout				
*E*_con_ days	1.23 ± 0.02	0.86 ± 0.11	0.70 ± 0.18	0.48 ± 0.08
*E*_max_ days	0.58 ± 0.03	0.83 ± 2.49	1.04 ± 0.94	0.45 ± 0.17
*H*	2.34 ± 0.33	1.0 ± 0.71	1.0 ± 0.25	1.0 ± 0.1
EC_50_ (AUC_0–24_/MIC)	0.45 ± 0.02	3.84 ± 21.44	2.10 ± 5.16	0.98 ± 1.40
EC_80_ (AUC_0–24_/MIC)	0.81 ± 0.04	15.36 ± 85.76	8.4 ± 20.64	3.92 ± 5.6
*r*^2^	>0.99	0.79	0.84	0.93

**Fig 5:**
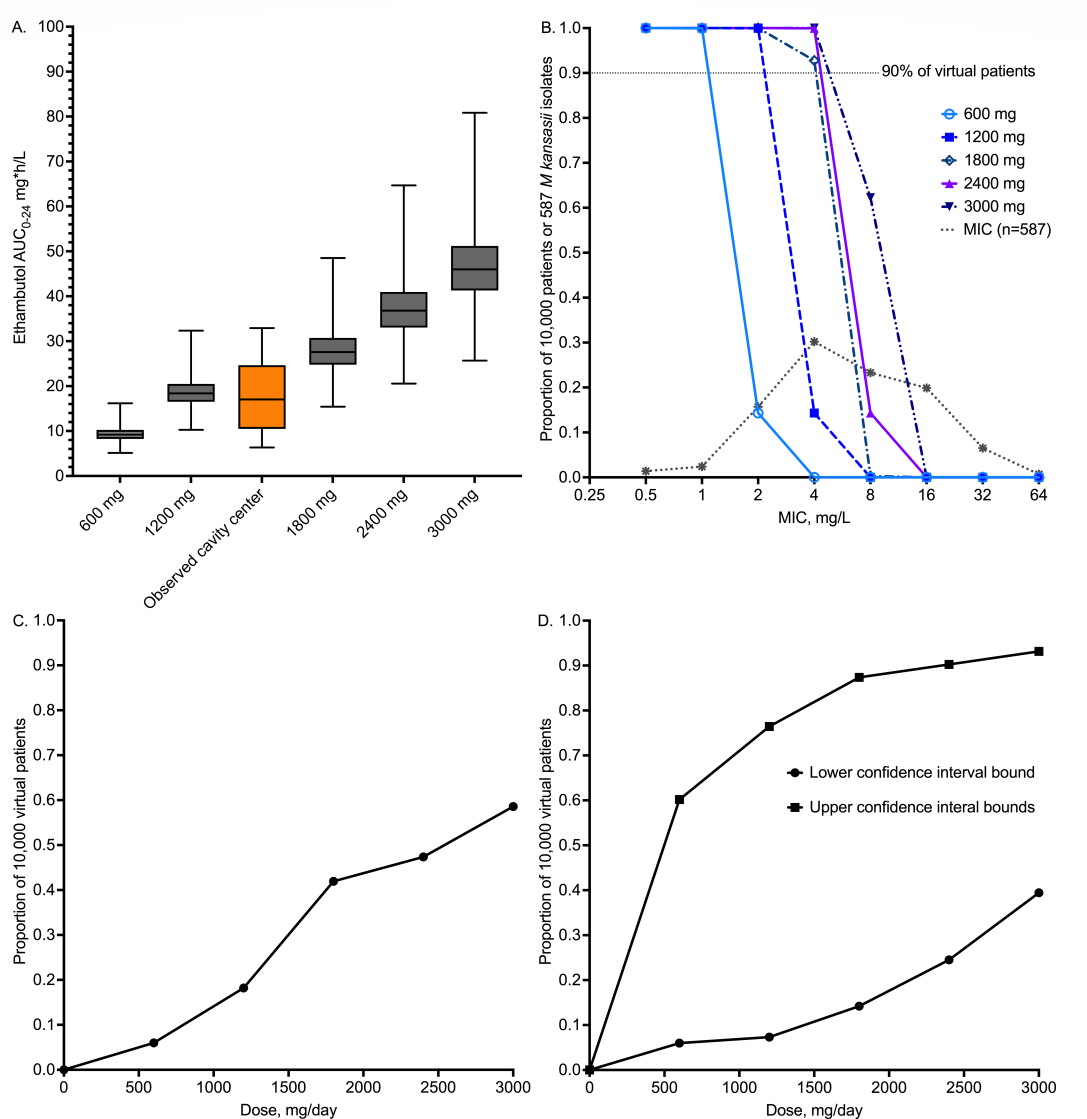
Monte Carlo experiments for *in silico* dose finding. All values were generated using the Monte Carlo method, in 10,000 virtual patients per dose. (**A**) Median 0–24 h area under the concentration-time curve (AUC_0–24_); error bars show maximum and minimum values. (**B**) Probability of target attainment of five ethambutol doses at each MIC. (**C**) Proportion of virtual subjects that achieve target exposure at cavity center for each dose. (**D**) Sensitivity analyses.

Next, we performed Monte Carlo experiments to identify the optimal ethambutol dose that achieves or exceeds the EC_80_ at the air-caseum interface, in MKC lung cavities, based PK parameters in lung cavities that we have published from prospective clinical studies (11, 18, 19). We did not examine the two important covariates of weight (including weight banding) and renal function but used the clearance for the average 64 kg weight in patients with MKC lung disease from Poland with normal renal function, assuming negligible ethambutol protein binding (18, 30, 39, 40). AUC results for the 1,200 mg/day dose in 10,000 virtual patients are shown in Fig. 5A, imposed on those we observed previously in lung cavities, demonstrating that PKs in our simulations were the same as those identified in the clinic. The probability of target attainment for doses of 600, 1,200, 1,800, 2,400, and 3,000 mg per day is shown in Fig. 5B, over the MIC distribution of all 587 isolates collated in Table 1. Figure 5B shows a susceptible-dose dependent PK/PD MIC breakpoint, from 1 mg/L at 600 mg, 2 mg/L at 1,200 mg, and 4 mg/L for ≥1,800 mg. It should be noted that currently, there is no established CLSI breakpoint for ethambutol against MKC, and the proposed breakpoints are solely based on the preclinical PK/PD data and modeling; therefore, they should be updated in the future as more information becomes available. Figure 5C shows how well each dose performs after taking an expectation over all MICs. Figure 5D is a sensitivity analysis based on taking either the upper or lower 95% confidence bound for target exposure. Even under the most generous assumptions (lower confidence bounds), the optimal dose (>90% of virtual patients) was 2,400 mg per day.

## DISCUSSION

First and foremost, here we show that ethambutol failed to achieve MKC microbial kill below *B*_0_. To put this into context, rifampin killed MKC below *B_0_* at all exposures tested in the HFS-MKC, and at *E*_max_ killed 5.0 log_10_ CFU/mL below *B*_0_ on day 21 of treatment. Similarly, isoniazid had a maximum kill below *B*_0_ of 2.57 log_10_CFU/mL. Thus, on a monotherapy basis, the least effective of the triad of first-line drugs is ethambutol. Figure 3A showed that the highest exposure tested in HFS-MKC reached full *E*_max_ on day 21, and since a drug cannot kill more than its *E*_max_, this means that testing higher exposures would not have resulted in any better microbial kill. Moreover, the clinical study by Zhang et al. (in Table 1) is instructive (28). In that study, the baseline ethambutol MIC distribution indicated ethambutol “resistance” (MIC > 4 mg/L) in 62% of patients versus rifampin resistance in 7%, and isoniazid resistance in 17% of MKC isolates (28). Despite the 62% ethambutol resistance in patient isolates, confirmed by whole-genome sequencing, 145 out of 191 patients (76%) achieved sustained sputum conversion at 2 months (28). Resistance to ethambutol was not associated with sputum conversion (odds ratio = 0.95, *P* = 0.884) (28). Thus, ethambutol resistance, as defined by both phenotypic and molecular evidence, which would have nullified the therapeutic effect of ethambutol, did not lead to failure of therapy. This clinical evidence, as well as failure to kill bacilli below *B*_0_ at *E*_max_ in the HFS-MKC, means ethambutol’s contributions to MKC microbial kill and sterilizing effect are meager, if any. This does not detract from the drug’s role in resistance suppression and in combination therapy. However, ethambutol is a better target for replacement by drugs that (i) kill MKC below *B*_0_ and (ii) suppress rifamycin, macrolide, and isoniazid resistance at the same time.

Ethambutol’s mutation frequency of 4.23% is a very narrow barrier to resistance emergence, defined as CFUs growing on agar supplemented with 3× ethambutol MIC. On the other hand, for all 587 isolates in Table 2, based on the proposed PK/PD MIC susceptibility breakpoint of 2 mg/L (for doses 1,200 mg) >80% would be defined as ethambutol resistant. In the HFS-MKC, there was concentration-dependent amplification of ethambutol resistance starting day 4, consistent with the antibiotic resistance arrow of time (41, 42). In Zhang et al.’s clinical study, cited above, among 46 patients with failed sputum culture conversion on rifampin plus isoniazid plus ethambutol therapy, comparison of MICs in paired serial isolates from *B*_0_ versus at 2 months found that while rifampin resistance increased from 2% to 9%, ethambutol resistance rose from 61% to a nearly universal 91% (28). Similarly, in the study by Ahn et al., spreadsheets of MICs of 13 wild-type isolates versus 14 isolates from patients who failed therapy or relapsed were reported (34). We compared the two MIC groups as ordinal data and found that MICs were much higher in patients who failed therapy or relapsed with either the Mann-Whitney test (*P* = 0.002) or the Kolmogorov-Smirnov test (*P* = 0.008). Taken together, all these data suggest that ethambutol resistance in MKC lung disease could occur rapidly and commonly. However, since ethambutol is administered in combination with two other drugs, which would suppress the ethambutol-resistance, more evidence needs to be gathered in the context of combination therapy.

If ethambutol were to be used for MKC lung disease, we found that even 3,000 mg would not be the optimal dose. In any case, such large doses pose the risk of ocular toxicity. The incidence of ethambutol-related ocular toxicity is dose-dependent: 1–2.5% for 15 mg/kg/day, 5–6% for 25 mg/kg/day, and 18% for 35 mg/kg/day (17, 43–46). In one study from North Texas, 8 out of 139 patients (6%) on daily ethambutol (175 mg/kg/week; mean 17.8 mg/kg/day) and 0 out of 90 patients (0%) on intermittent ethambutol (75 mg/kg/week; 10.7 mg/kg/day equivalent) developed ocular toxicity, strongly suggesting cumulative AUC-based toxicity (44). The severity of the visual loss is also dose dependent, with a visual loss of 20/40 that resolves in 1.5 months at doses below 30 mg/kg versus 20/200 and resolving after 5 months at doses higher than 30 mg/kg (46). The Monte Carlo experiments-derived doses should be interpreted in that context; as an example, taking the weight reported in MKC lung disease female patients in Poland (30), doses of 1,200 mg/day correspond to 19 mg/kg/day, 1,800 mg/day would be 29 mg/kg, and 3,000 mg would be 48 mg/kg/day. Thus, likely most of the doses would be associated with a high risk of ocular toxicity versus the poor benefit of a drug that does not kill *M. kansasii* below *B*_0_ or achieve target attainment in greater than 90% of patients.

Finally, we have described previously “wobbling” of PK/PD driver and potency (EC_50_ and EC_80_) between sampling days in slow-growing mycobacteria such as *M. avium* complex and TB (47–49). Here, we would like to add MKC to these mycobacteria with “wobbling” of the PK/PD driver. The reasons for this are unclear. However, what is certain is that this leads to the logical problem of which sampling day to use for calculating the target exposure in preclinical studies.

Our study has several limitations. First, it could be argued that higher exposures than we used in R1 to R6 should have been investigated. However, those exposures would have been supraphysiological and above what is achieved at the center of lung cavities in patients on high ethambutol doses (19). In addition, the exposures tested here reached *E*_max_, which means that the drug would have saturated its target, bacterial arabinosyl transferase, and increasing the exposures would not have improved the effect (50–52). Second, we did not perform dose-fractionation studies. Third, we examined only a single isolate of MKC, whereas four to five are required for robust PK/PD target exposure derivation. These two factors would have increased resources and expenses; however, MKC lung disease has been the most ignored by funders and investigators among non-tuberculous mycobacteria. Third, we did not examine ethambutol’s primary role of suppressing resistance to companion drugs, but instead what it contributes to microbial kill. Finally, elsewhere, we have defined quality criteria for hollow fiber model system studies for TB and *M. avium-*complex but have not yet developed those for HFS-MKC (49, 53). The tool for MKC quality controls still needs to be defined for preclinical PK/PD models.

In summary, here we found that ethambutol efficacy in the HFS-MKC is meager, if any. Monte Carlo experiments demonstrated that doses with good target attainment would be high, in a range likely to be toxic to patients. Emergence of ethambutol resistance to the therapy occurs relatively rapidly. In short, the risk of toxicity versus no microbial kill benefit, as shown in HFS-MKC, might need to carefully reconsider ethambutol with respect to MKC lung disease by finding drugs that have both microbial kill effect as well as suppressing resistance to companion drugs.

## MATERIALS AND METHODS

### Literature search strategy

Our rationale for the literature search was to identify ethambutol MIC distributions in MKC clinical isolates from different geographic locations, for use in Monte Carlo experiments with virtual subjects. We performed a PubMed search using the MeSH headings “*Mycobacterium kansasii* AND MIC AND ethambutol” for MIC distributions. We excluded studies that tested fewer than 10 isolates or tested a concentration range of less than five MIC dilutions or did not report the number or proportion of isolates at each MIC. We did not exclude any publications by language. Two investigators performed the literature search.

### Bacteria, cell line, drug, and other supplies

MKC standard laboratory strain (ATCC#12478) was used in all reported studies. For bacterial culture, we used Middlebrook 7H9 broth and Middlebrook 7H10 agar, both supplemented with 10% oleic acid-albumin-catalase-dextrose (OADC). The human monocyte-derived THP-1 cell line (ATCC TIB-202) was grown in RPMI-1640 medium supplemented with heat-inactivated 10% fetal bovine serum (FBS). In the HFS-MKC studies, the circulating medium was RPMI-1640 supplemented with 2% FBS. Ethambutol was purchased from BOC Sciences (NY, USA), cellulosic hollow fiber cartridges from FiberCell Systems Inc. (MD, USA), and the MGIT liquid culture system and EpiCenter software were purchased from Becton and Dickinson, USA.

### Ethambutol MIC and mutation frequency

Ethambutol MIC to MKC was determined using the broth microdilution method (54). To determine the ethambutol mutation frequency, agar supplemented with 3× MIC concentration was inoculated with 0.2 mL of logarithmic phase growth MKC with an intended initial bacterial burden of approximately 4.5 log_10_ CFU/mL. The total volume used for the mutation frequency was 4 mL spread on 20 agar plates.

### Ethambutol PK/PD in the HFS-MKC

The detailed description of the HFS in general and to perform the PK/PD studies with MKC has been published previously (4, 6–8, 55, 56). We tested eight different ethambutol doses, including two non-treated control HFS-MKC units. To put this in clinical context, a previous study by Peloquin et al. (57) reporting ethambutol PK in healthy males and females in a randomized trial with a 25 mg/kg daily dose (mean dose of 1,936 ± 343 mg), the peak concentration in serum was observed as 4.5 ± 1.0 mg/L and half-life of approximately 3.3 ± 0.8 h, assuming 100% bioavailability. However, in the present study, the dilution rate in the HFS-MKC was set to mimic the ethambutol PK as achieved in TB patients’ lung cavities, mimicking six low and high doses of ethambutol (19, 21). The HFS-MKC mimicked the free (unbound) concentrations.

To briefly describe the study design, the THP-1 cells were co-cultured with log-phase growth MKC cultures for 4 h at a multiplicity of infection of 1:1. Next, the infected THP-1 cells were washed two times with warm RPMI-1640 to remove the extracellular bacteria, and 20 mL of the THP-1 cell suspension was inoculated into the peripheral compartment of each of the eight HFS-MKC units, where the circulating medium was RPMI-1640 supplemented with 2% FBS to maintain the cell viability. The HFS-MKC units were treated with different ethambutol doses, once daily over 1 h using computer-controlled syringe pumps. First-order kinetics can be used to explain the drug diffusion into the peripheral compartment, that is, across the hollow fibers into and out of the extra-capillary space. To determine the PK of each ethambutol dose, the central compartment of each HFS-MKC unit was sampled pre-dose followed by 2, 5, 8, 12, 18, and 23.5 h post-drug infusion. For the PD of ethambutol in the HFS-MKC, samples were collected from the peripheral compartment of each HFS-MKC unit on days 4, 7, 14, and 21. The number of viable THP-1 cells was determined manually and using an automated cell counter (Scepter 2.0 Cell Counter, Millipore Sigma). After the samples were washed to remove carryover drug, THP-1 cell lysis was performed to release the intracellular bacterial, followed by 10-fold serial dilution in normal saline to determine the bacterial burden using the Middlebrook 7H10 agar supplemented with 10% OADC. The same samples were also cultured on agar supplemented with 24 mg/L ethambutol (3× MIC) to determine the drug-resistant subpopulation at each time point. The CFU/mL was recorded after 10 days of incubation at 37°C. As a second PD method, we used the MGIT liquid culture system where 500 µL of the washed and undiluted sample from each HFS-MKC unit was inoculated into the MGIT tubes. EpiCenter software was used to record the growth units and time-to-positive in each sample.

### Measurement of ethambutol in HFS-MKC samples

The method to measure the ethambutol concentration in the experimental samples has been published previously and was used without any modification (4, 19, 21, 58). Briefly, ethambutol and its internal standard (IS), ethambutol-d10, were purchased from CDN Isotopes (Quebec, Canada) and Sigma-Aldrich (St. Louis, MO, USA), respectively. LC-MS/MS analysis was performed using Waters Acquity UPLC coupled with Waters Xevo TQ mass spectrometer. Separation was achieved by injecting 10 µL of sample on a Waters Acquity UPLC HSS T3 column (50 × 2.1 mm; 1.8 µm) using a binary gradient. Solvents were: (i) 0.1% aqueous formic acid, and (ii) 0.1% formic acid in methanol. Samples were diluted 1:10 with an IS solution containing ethambutol-d10. The transitions used were *m/z* 205–116 (ethambutol), and *m/z* 215–123 (ethambutol-d10). The flow rate was 0.2 mL/min, and the total run time was 6 min. The between-day percentage coefficient of variation (%CV) for analysis of low and high (in parenthesis) quality controls was 14% (4%). The intraday %CV for rifampin was 14% (2%). The lower limit of ethambutol quantitation was 0.01 µg/mL.

### Data analysis

MIC distributions were fitted to a Gaussian function for each study, as well as when all isolates were combined. In the HFS-MKC, total bacterial burden was modeled versus PK/PD exposure using the inhibitory sigmoid *E*_max_ model (59, 60). Ethambutol-resistant subpopulation versus PK/PD exposures were modeled using a quadratic function that is an integral part of the antibiotic resistance arrow of time model (41, 61).

### Monte Carlo experiments

Monte Carlo experiments were performed in ADAPT 5, with steps discussed extensively in our prior publications (62–64). Briefly, PK parameter inputs into subroutine prior were from published population PK studies, including penetration into lung cavities (11, 18, 19). We did not examine for the two important covariates of weight (including weight banding) and renal function, but used the clearance for the average 64 kg weight in patients with MKC lung disease from Poland with normal renal function, assuming negligible ethambutol protein binding (18, 30, 39, 40). We assumed a two-compartment model with the following parameters and variances (%): total clearance of 39.7 L/h (inter-individual variance [IIV] = 34%), central volume of 90 L (IIV = 40%), absorption constant (*K*_a_) of 0.498 per hour (IIV = 43%), intercompartmental clearance 33.8 L/h (IIV = 40%), and peripheral volume of 642 L (IIV = 40%). The blood compartment in Fig. 2B represents the central compartment, linked to the different cavity positions by a dynamical sink model-based gradient:

In the past, we wrote and published (19, 21) the dynamical sink model described by the equation:


$$\frac{dD}{ds}=\alpha \frac{D}{D+K}-(\mu +u_{s})D.$$

$$u_{s}\left(s\right)=X_{max}\left(\frac{1}{1+e^{\nu \left(c_{0}-s-r\right)}}+\frac{1}{1+e^{-\nu \left(c_{0}+r-s\right)}}-1\right)$$

where *D* is drug concentration, *s* is distance in centimeters from blood vessel, the function, $u_{s}\left(s\right)$, captures the changing landscape of the cavity tissues starting from blood to the are-caseum interface at cavity center, $X_{max}$ is the cavity potential energy, $c_{0}$ is the center of the sink, r is the radius of the sink. Here, we modeled only concentrations at the center of the sink.

We examined the ethambutol oral doses of 600, 1,200, 1,800, 2,400, and 3,000 mg for the ability to achieve or exceed the PK/PD target exposure at the center of lung cavities. The PK/PD target exposure was identified in HFS-MKC studies. We generated ethambutol AUCs in the lung cavities of 10,000 virtual subjects for each dose. The probability of target attainment at each of the MICs in 587 isolates identified in our literature search was calculated. The cumulative fraction of response was calculated by taking an expectation over all the MICs.

## Data Availability

Data can be made available upon request.
